# Reduced Regional Cerebral Blood Flow Measured by ^99m^Tc-Hexamethyl Propylene Amine Oxime Single-Photon Emission Computed Tomography in Microgravity Simulated by 5-Day Dry Immersion

**DOI:** 10.3389/fphys.2021.789298

**Published:** 2021-11-22

**Authors:** Laurent Guillon, Marc Kermorgant, Thomas Charvolin, Fabrice Bonneville, Marie-Pierre Bareille, Emmanuelle Cassol, Arnaud Beck, Marie Beaurain, Patrice Péran, Jean-Albert Lotterie, Anne Pavy-Le Traon, Pierre Payoux

**Affiliations:** ^1^Department of Nuclear Medicine, Toulouse University Hospital, Toulouse, France; ^2^INSERM UMR 1297, Institute of Cardiovascular and Metabolic Diseases (I2MC), Toulouse University Hospital, Toulouse, France; ^3^Department of Neuroradiology, Toulouse University Hospital, Toulouse, France; ^4^INSERM URM 1214, Toulouse NeuroImaging Center (ToNIC), Toulouse University Hospital, Toulouse, France; ^5^Institute for Space Medicine and Physiology (MEDES), Toulouse, France; ^6^Department of Neurology, Toulouse University Hospital, Toulouse, France

**Keywords:** HMPAO, regional cerebral blood flow, microgravity, dry immersion, thigh cuffs, DI5-CUFFS

## Abstract

Microgravity induces a cephalad fluid shift that is responsible for cephalic venous stasis that may increase intracranial pressure (ICP) in astronauts. However, the effects of microgravity on regional cerebral blood flow (rCBF) are not known. We therefore investigated changes in rCBF in a 5-day dry immersion (DI) model. Moreover, we tested thigh cuffs as a countermeasure to prevent potential microgravity-induced modifications in rCBF. Around 18 healthy male participants underwent 5-day DI with or without a thigh cuffs countermeasure. They were randomly allocated to a control (*n*=9) or cuffs (*n*=9) group. rCBF was measured 4days before DI and at the end of the fifth day of DI (DI5), using single-photon emission computed tomography (SPECT) with radiopharmaceutical ^99m^Tc-hexamethyl propylene amine oxime (^99m^Tc-HMPAO). SPECT images were processed using statistical parametric mapping (SPM12) software. At DI5, we observed a significant decrease in rCBF in 32 cortical and subcortical regions, with greater hypoperfusion in basal ganglia (right putamen peak level: *z*=4.71, *p*_uncorr_<0.001), bilateral occipital regions (left superior occipital peak level: *z*=4.51, *p*_uncorr_<0.001), bilateral insula (right insula peak level: 4.10, *p*_uncorr_<0.001), and bilateral inferior temporal (right inferior temporal peak level: 4.07, *p*_uncorr_<0.001). No significant difference was found between the control and cuffs groups on change in rCBF after 5days of DI. After a 5-day DI, we found a decrease in rCBF in cortical and subcortical regions. However, thigh cuffs countermeasure failed to prevent hypoperfusion. To date, this is the first study measuring rCBF in DI. Further investigations are needed in order to better understand the underlying mechanisms in cerebral blood flow (CBF) changes after exposure to microgravity.

## Introduction

Exposure to microgravity has detrimental effects on human physiology, such as muscle atrophy, bone demineralization, sensorimotor and cardiovascular deconditioning, and immune, hormonal, and metabolic changes (Michel et al., 1976; West, 2000). Body fluid redistribution begins in the first hours of space flight. This so-called cephalad fluid shift is responsible for cephalic venous stasis, characterized by dilation of the jugular vein and facial oedema. This phenomenon is mainly due to loss of the cranial-to-caudal flow gradient induced by weightlessness (Parazynski et al., 1991; Arbeille et al., 2001). During long-duration spaceflights, the cephalad fluid shift observed in astronauts may increase intracranial pressure (ICP), as suggested by the assessment of optic nerve sheath diameter (ONSD) by ultrasound and MRI (Kramer et al., 2012; Sirek et al., 2014) and cerebral hemodynamics seem to be modified. However, these mechanisms are not fully understood.

Indirect assessment of cerebral blood flow (CBF) by transcranial Doppler ultrasound of the middle cerebral artery has revealed a decrease in cerebral vascular resistance (CVR) and an increase in CBF during the first days of space flight, after which these parameters normalize (Arbeille et al., 2001). Cerebral autoregulation is the mechanism that maintains CBF relatively constant, despite change in cerebral perfusion pressure (CPP). Previous studies have shown that cerebral autoregulation is preserved or even improved after short-term exposure to microgravity, whereas cerebral autoregulation was impaired after long-term exposure (Kermorgant et al., 2020). Nevertheless, the mechanisms behind modifications in CBF, CVR, and cerebral autoregulation after exposure to weightlessness have not yet been clearly elucidated.

**Figure 1:**
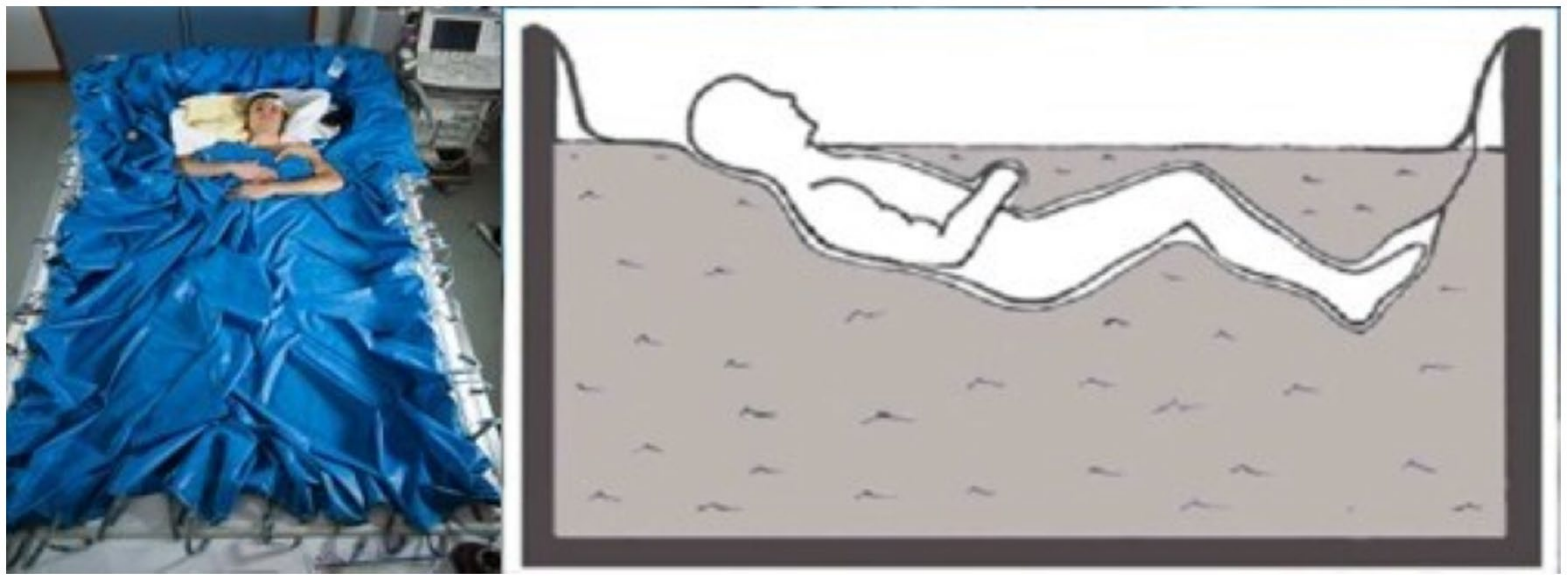
Illustration of dry immersion (DI). Participants are immersed in a half-seated position, up to the neck and separated from the water by a waterproof fabric (Photo MEDES/E. GRIMAULT).

Different methods are used to study microgravity on Earth. Head-down bed rest (HDBR) is the most used method and it induces most of the effects on the human body observed during space flight, including cephalad fluid shift (Hargens and Vico, 2016). Dry immersion (DI) consists of immersing a subject into thermoneutral water covered with a waterproof fabric, the subject being “free suspended” in the water bath. DI reproduces most of the change observed during space flight, and even more rapidly and more intense than with HDBR (Navasiolava et al., 2011; Figure 1). Few studies have measured regional (r) CBF in humans after exposure to simulated microgravity. Guell et al. (1982) found in healthy volunteers who underwent −4° HDBR for 7days, an increase in regional cerebral blood flow (rCBF; measured by ^133^Xe inhalation method) after 6h, but returned to the baseline state at 72h (Guell et al., 1982). No study has so far measured rCBF both during spaceflight and microgravity analogs such as DI.

**Figure 2:**
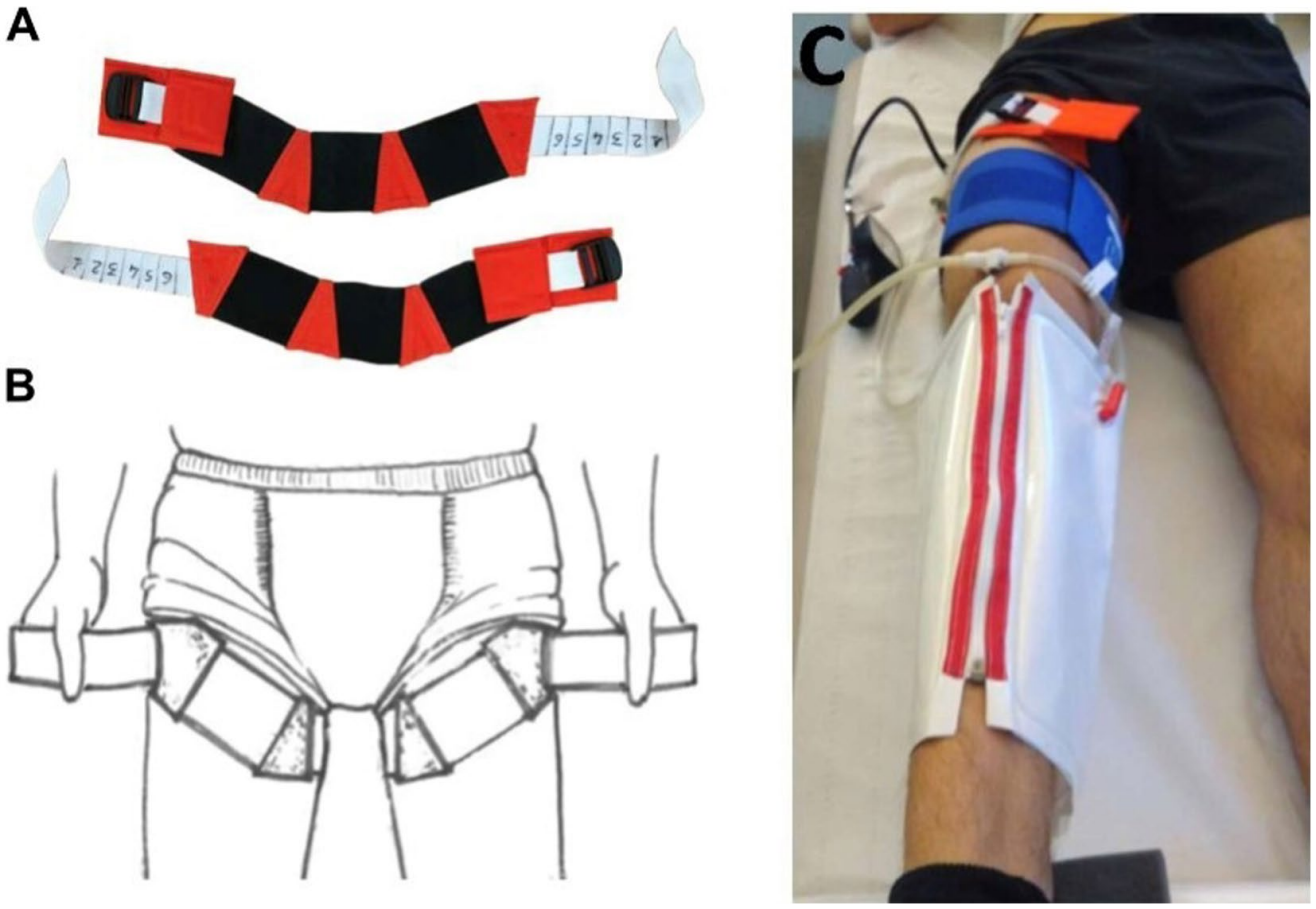
Illustration of thigh cuffs. **(A)** Thigh cuffs are elastic strips that can be adjusted to the size of the thigh with a clamping segment (white segment); **(B)** Thigh cuffs are worn on the upper thigh. **(C)** Individual adjustment of thigh cuffs with plethysmography to apply a 30-mmHg pressure on the upper thigh, performed at baseline data collection (BDC)-2 (Photo MEDES).

Thigh cuffs are elastic strips that are designed to have the same effects on lower-limb distensibility as a counterpressure of about 30mmHg. These elastic strips, used by Russian cosmonauts, are effective against the cephalad fluid shift by trapping the venous volume in the lower limbs. Thigh cuffs are generally worn by cosmonauts during the day and removed at night for comfort reasons (Pavy-Le Traon et al., 2001; Figure 2).

The aim of the present study was to investigate possible changes in rCBF using DI as a ground-based model of microgravity. A second objective was to test whether thigh cuffs can serve as a countermeasure, limiting any changes in rCBF, by restricting the cephalad fluid shift and potential increase in ICP.

## Materials and Methods

### Participants

Twenty healthy men were recruited. Two withdrew before the 4days of baseline data collection (BDC) for reasons unrelated to the protocol. A total of 18 participants were therefore included in the study and randomly allocated to either a control or a cuffs group (9/9 split). All participants were informed about the experimental procedures and gave their written consent. The experimental protocol was conducted in accordance with the standards set by the Declaration of Helsinki and approved by the local ethics committee (CPP Est III: 2 October 2018, no. ID RCB 2018-A01470-55) and French health authorities (ANSM: 13 August 2018). ClinicalTrials.gov identifier: NCT03915457.

### General Protocol

The experiment (DI5-CUFFS) was an integrative study carried out at the MEDES Space Clinic in Toulouse (France) from 19/11/2018 to 23/03/2019. The experimental protocol consisted of 4days of ambulatory BDC before DI (BDC-4 to BDC-1), 5days (120h) of DI (DI1–DI5), and 2days of ambulatory recovery (R0, R+1, and R+2 morning).

A week before the beginning of the protocol, participants went to MEDES for a Pre-DI thigh muscle biopsy and resting metabolic rate measurement.

Participants into the cuffs group wore the thigh cuffs throughout the 5days of DI, from 10AM to 6PM on DI1, and from 8AM to 6PM on DI2-DI5. Calf plethysmography, performed in the supine position at BDC-2, was undertaken to adjust the cuffs to each participant. At DI1, thigh cuffs were placed on subjects immediately prior to DI onset at 10AM.

The general protocol for DI was implemented according to the methodology described elsewhere (Friston et al., 1991). Two participants, one control and one cuffs, underwent DI simultaneously in the same room, in two separate baths (except for two participants, one cuffs and one control, who were each alone in the room). Thermoneutral water temperature was continuously maintained (32.5–33.5°C). Lights were switched off from 11PM to 7AM. Daily hygiene, weighing and some specific measurements required exit from the bath. During these out-of-bath periods, participants maintained the −6° head-down position. Total out-of-bath supine time for the 120h of immersion was 9.7±1.3h. On DI1-DI4, out-of-bath time was 1.1±0.6h/day. On DI5, out-of-bath time was 5.3±1.1h, owing to a muscle biopsy in the right thigh and encephalic and spinal MRI. Otherwise, during DI, participants remained immersed in a half-seated position for all activities and were continuously subjected to video monitoring. Bodyweight, blood pressure, heart rate (HR), and tympanic body temperature were measured daily. Water intake was fixed at 35–60ml/kg/day. Within these limits, water intake throughout the protocol was *ad libitum* and quantified. The menu for each experimental day was identical for all participants, and dietary intake was individually tailored and controlled during the study. Measurements of HR and arterial blood pressure were performed with an automatic device twice a day (morning and evening). VO_2_max was measured in the evening of B-2 and R0. Percent change in plasma volume on DI-1-evening, DI-3-morning, DI-5-morning, DI-5-evening, R0-morning, and baseline (DI-1-morning before the onset of immersion) was estimated using Hb and Hct count (Dill and Costill formula).

Daily questionnaires were proposed to subject each morning and evening from B-1 to R0. Visual analog scale 0–10 was used to assess General discomfort, Back pain, Quality of night sleeping, and Discomfort at thigh level. Scoring scheme of 0–5 was used for “Fluid shift” complaints-face swelling sensation, nasal congestion, and impaired vision.

### Single-Photon Emission Computed Tomography Acquisitions

^99m^Tc-hexamethyl propylene amine oxime (^99m^Tc-HMPAO) is a lipophilic radiopharmaceutical used for measuring rCBF. The radio-labelled compound was prepared from a commercial kit (Cerestab™; GE Healthcare, Norway), mixed with sodium-(^99m^Tc)-pertechnetate and diluted in a saline solution (0.9% sodium chloride). At BDC-4, 261±8MBq of ^99m^Tc-HMPAO were intravenously administered, within 3h of preparation. Before and after the injection, participants were isolated from sensory stimulations in a dark and quiet room, wearing earplugs and a sleep mask for 10min. The ^99m^Tc-HMPAO injection performed at BDC-4 was conducted in a half-seated position, so that participants were in a similar position to that at R0 when, just before the end of DI, 263±10MBq were injected, while participants were immersed in the bath. Both injections for all the subjects took place in the morning, between 9 and 11AM.

Single-photon emission computed tomography (SPECT) acquisitions were performed on a dual-head hybrid camera (SymbiaT6; Siemens Healthcare, Erlangen, Germany) equipped with a low-energy high-resolution collimator. The energy window was 140keV±7.5% (with additional low energy window for scatter correction). Acquisition parameters for SPECT were: 60 projections over 180°, with 30s per projection (matrix: 128×128, zoom 1.78). To perform attenuation correction, a brain computed tomography (CT) scan was also acquired with the following parameters: 110kV, 50mAs, and collimation 6×2mm. Iterative reconstruction was performed with a flash3D algorithm (12 iterations, eight subsets, and 8-mm Gaussian filter). Images with scatter and CT-attenuation corrections were then generated. Any decrease in radioactivity was corrected during analysis with statistical parametric mapping (SPM12) software, by applying a weighting factor depending on the radioactivity period of ^99m^Tc for each acquisition.

### Statistical Analysis

Single-photon emission computed tomography images were processed using SPM12 software (Evans et al., 1993), implemented in MATLAB (MathWorks, Sherborn, MA, United States). SPM combines the general linear model and theoretical Gaussian fields to make statistical inferences about regional effects. All SPECT images were realigned and normalized to a standard template in MNI space using SPM12 (Wilkerson et al., 2005), then smoothed with a Gaussian kernel filter of 8mm at full width and half maximum. We compared rCBF at BDC-4 compared to R0 for all the subject together, using a paired *t*-test, considering that our data are normally distributed. We also compared rCBF between the cuffs and control group at BDC-4 and at R0, and the change in rCBF during DI between the cuffs and control groups, using a two-sample *t*-test. We tested the null hypothesis that the voxel-to-voxel contrast is zero. For all the tests, we chose an uncorrected threshold *p*_uncorr_<0.001 with an extended threshold of 100 voxels. From the SPM12 results, we extracted a statistical parametric map in t_score_, overlaid on a MRI template that represents the result of the change in rCBF at R0 compared with BDC-4, for an uncorrected threshold *p*_uncorr_<0.001 with an extended threshold of 100 voxels.

General hemodynamic parameters (heart rate, systolic, diastolic, and mean arterial blood pressure) were expressed as mean±SD and 95% CI of the mean.

## Results

### Group Characteristics

Baseline group characteristics are detailed in Table 1.

**Table 1:** Baseline group characteristics at BDC-2.

	All (*n*=18)	Control (*n*=9)	Cuffs (*n*=9)
Age (years)	34.0±5.5	33.9±7.1	34.1±3.7
Right-handed	16	8	8
Height at selection (cm)	178±6	176±6	180±4
Weight (kg)	74.1±8.0	73.9±7.5	74.3±8.8
BMI (kg/m^2^)	23.3±1.8	23.9±1.7	22.7±1.8
VO_2_ max (ml/min/kg)	46.7±6.9	46.5±8.1	46.9±5.8
Morning T (°C)	36.4±0.4	36.4±0.3	36.4±0.5

### rCBF Measurement

**Figure 3:**
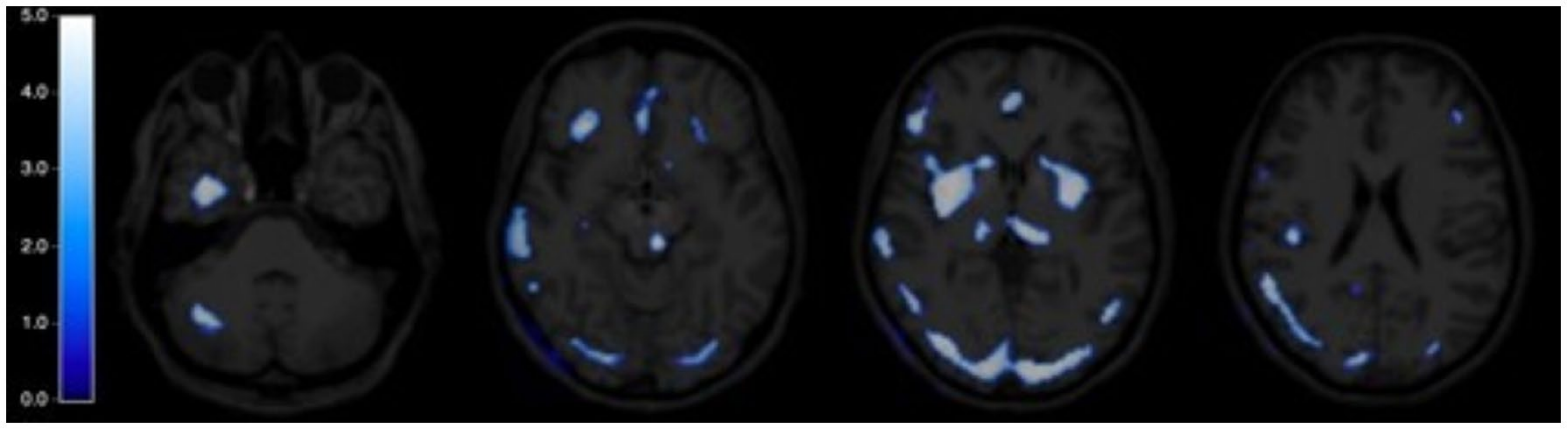
Statistical parametric map of regional cerebral blood flow (rCBF) after DI. Cerebral map expressed in t_score_ of the negative change in rCBF at R0 compared with BDC-4, paired *t*-test, *p*_uncorr_<0.001, extent threshold>100 voxels.

Regional cerebral blood flow was significantly reduced in cortical and subcortical regions at R0, compared with BDC-4, with a significance threshold of *p*_uncorr_<0.001 and an extended threshold of 100 voxels. Around 32 cortical and subcortical regions that were significantly less perfused at R0 than at BDC-4 were highlighted, the decrease in rCBF being greater in basal ganglia (right putamen peak level: *z*=4.71, *p*_uncorr_<0.001), bilateral occipital regions (left superior occipital peak level: *z*=4.51, *p*_uncorr_<0.001), bilateral insula (right insula peak level: 4.10, *p*_uncorr_<0.001), and bilateral inferior temporal (right inferior temporal peak level: 4.07, *p*_uncorr_<0.001; Table 2; Figure 3).

**Table 2:** Negative change in regional cerebral perfusion after DI.

	*z*_score_ peak level	*t*_score_ peak level	Number of voxels in cluster
Basal ganglia			
Left caudate	3.39	4.13	680
Left putamen	4.09	5.46	680
Right caudate	3.80	4.88	3,213
Right putamen	4,71	6.92	3,213
Brainstem			
Left midbrain	4.16	5.61	1,006
Right midbrain	*ns*		
Cerebellum			
Left cerebellum	3.78	4.82	120
Right cerebellum	3.93	5.12	551
Cortex			
Cingulate			
Left middle cingulate gyrus	*ns*		
Left anterior cingulate gyrus	*ns*		
Left posterior cingulate gyrus	*ns*		
Right middle cingulate gyrus	3.55	4.40	105
Right anterior cingulate gyrus	3.47	4.27	269
Right posterior cingulate gyrus	3.97	5.20	130
Frontal			
Left superior frontal gyrus	*ns*		
Left medial orbital gyrus	3.45	4.24	144
Left middle frontal gyrus	3,72	4,72	100
Left posterior orbital gyrus	3.54	4.40	144
Left superior frontal gyrus medial	*ns*		
Right superior frontal gyrus	3.57	4.44	269
Right medial orbital gyrus	3.42	4.20	3,213
Right middle frontal gyrus	*ns*		
Right posterior orbital gyrus	3.92	5.09	3,213
Right superior frontal gyrus medial	3.57	4.44	269
Insula			
Left insula	4.09	5.46	680
Right insula	4.10	5.48	3,213
Occipital			
Left inferior occipital gyrus	4.35	6.02	6,570
Left middle occipital gyrus	4.35	6.02	6,570
Left superior occipital gyrus	4.51	6.42	6,570
Right inferior occipital gyrus	4.38	6.09	6,570
Right middle occipital gyrus	4.38	6.09	6,570
Right superior occipital gyrus	4.38	6.09	6,570
Parietal			
Left angular gyrus	*ns*		
Left postcentral gyrus	*ns*		
Right angular gyrus	3.17	3.78	157
Right postcentral gyrus	3.74	4.75	157
Temporal			
Left fusiform gyrus	3.61	4.52	121
Left inferior temporal gyrus	4.06	5.40	201
Right fusiform gyrus	*ns*		
Right inferior temporal gyrus	4.07	5.41	308
Thalamus			
Left thalamus	4.46	6.30	1,006
Right thalamus	3.67	4.62	1,006

Negative change in regional cerebral perfusion at R0 compared with BDC-4 in all the 18 participants, in maximum z_score_, maximum t_score_, number of significant voxels per cluster; *p*_uncor_<0.001 and extent threshold>100 voxels.

There was no significant difference in rCBF between the cuffs and control groups at BDC-4 and R0 (*p*_uncorr_<0.001 and extended threshold of 100 voxels).

There was no significant difference in the change in rCBF at R0 compared with BDC-4 between the cuffs and control groups (*p*_uncorr_<0.001 and extended threshold of 100 voxels).

### Blood Pressure and Heart Rate

Heart rate, systolic blood pressure (SBP), diastolic blood pressure (DBP), and mean blood pressure (MBP) were not significantly changed after DI, both in the control and cuffs groups (Table 3).

**Table 3:** Hemodynamic parameters.

	All (*n*=18)		Control (*n*=9)		Cuff (*n*=9)	
	BDC-2	R0	BDC-2	R0	BDC-2	R0
HR (bpm)	58±7 (54–61)	58±8 (55–62)	58±8 (53–64)	57±9 (51–63)	57±6 (53–60)	60±7 (55–65)
SBP (mmHg)	116±10 (111–121)	117±10 (113–121)	117±10 (110–124)	114±10 (107–120)	115±11 (108–123)	120±8 (115–126)
DBP (mmHg)	68±7 (65–71)	67±8 (64–71)	68±9 (62–73)	66±7 (62–71)	68±5 (65–72)	68±9 (63–74)
MBP (mmHg)	86±7 (83–89)	85±7 (82–88)	86±8 (80–91)	83±7 (78–88)	86±5 (83–89)	87±7 (83–92)

Measurements for heart rate (HR), systolic blood pressure (SBP), diastolic blood pressure (DBP), and mean blood pressure (MBP), before (BDC-2) and after (R0) DI. All measurements were performed in the morning. Mean±SD. Values in parentheses represent 95% CI of the mean.

### Daily Questionnaires, VO_2_ Max, and Plasma Volume

Results about daily questionnaires, VO_2_ max measurement, and plasma volume are detailed in Robin et al. (2020).

There was no significant correlation between change in plasma volume, scoring of general discomfort, back pain, quality of night sleeping, discomfort at thigh level, face swelling sensation, nasal congestion, or impaired vision with decrease in rCBF at R0.

## Discussion

### Decrease in rCBF After a 5-Day DI

After 5days of DI, we observed a significant decrease in rCBF in 32 cortical and subcortical regions. No previous study had measured rCBF in humans after DI. A study in HDBR measuring rCBF with the ^133^Xe inhalation method found an initial increase at 6h, but no difference at 72h (Guell et al., 1982). Some studies measuring rCBF have been performed in animals. Consistently with our results, Wilkerson et al. (2005) demonstrated in a 2-week head-down tail suspension study performed in rats, a decrease in rCBF in 21 cortical and subcortical regions, measured with [^14^C]-IPIA autoradiography, the decrease being more intense in the basal ganglia (Wilkerson et al., 2005).

We measured a decrease in rCBF in many cortical and subcortical regions, mostly in the basal ganglia, insula, occipital, and inferior temporal cortex. These regions are involved in many brain functions, such as the control of movements and equilibrium, sensorimotor, vegetative, cognitive, and limbic functions, those are impaired after microgravity exposure (Hasser and Moffitt, 2001; Koppelmans et al., 2017; Lacquaniti et al., 2017; Van Ombergen et al., 2017). Moreover, a greater reduction in gray matter volume (measured by MRI) in multiple brain regions, including regions located in the bilateral frontal lobes, temporal poles, insula, and right hippocampus was observed during a 30-day HDBR study (Li et al., 2015).

### Hypothesis on the Mechanisms Which May Decrease rCBF

As the cranial box is rigid and inextensible, and intracranial content is not compressible, ICP depends on three parameters: craniospinal elastance, resistance to cerebrospinal fluid flow, and brain blood volume (Tameem and Krovvidi, 2013). Although ICP has never been directly measured during long exposure to microgravity in humans, indirect evaluation methods (measurement of ONSD) suggest an increase in ICP favored by the cephalad fluid shift (Hargens and Watenpaugh, 1996; Kramer et al., 2012). However, the magnitude of a possible increase in ICP during space flights and its precise underlying mechanisms remain unclear.

Cerebral perfusion pressure is the result of mean arterial pressure (MAP) and ICP, according to the equation CPP=MAP – ICP (Partington and Farmery, 2014). Consistently with our findings, studies have shown that MAP does not seem to vary significantly in studies in HDBR (Arbeille et al., 2001) and after 3-day DI (Ogoh et al., 2017).

According to Poiseuille’s law, CVR depends on cerebral vessel diameter. CBF depends on CPP and CVR, according to the equation CBF=CPP/CVR. When CPP decreases or CVR increases, that leads to a decrease in CBF. CPP is directly correlated to ICP; When ICP increases, CPP is maintained by an increase in blood pressure up to a certain limit. There is no direct measurement of ICP in microgravity in humans. However, direct measurements performed in animals (Krotov et al., 1994) and indirect measurements in humans (Kramer et al., 2012; Kermorgant et al., 2017) argue in favor of a moderate elevation in ICP. During 3-day DI, Kermorgant et al. (2017) showed an increase in ONSD of about 30%, as measured with ultrasound (Pre-DI: 4.64±0.40mm; DI3: 6.01±0.49mm; *p*<0.001; Kermorgant et al., 2017). In DI5-CUFFS, Kermorgant et al. (2021) have found a significant increase of ONSD after 5days of DI by 20% in the control group and 14% in the cuffs group (Kermorgant et al., 2021). These ONSD values are equivalent to an elevation of ICP around 20mmHg, the normal range being between 7 and 15mmHg (Geeraerts et al., 2008). It, therefore, seems unlikely that a moderate elevation in ICP during DI would exceed the adjustment capacities of CPP.

During HDBR studies, Doppler measurements showed an increase in CVR and a decrease in CBF during the first week, after which these parameters returned to baseline values (Frey et al., 1993; Arbeille et al., 2001; Sun et al., 2002). After 3-day DI, Ogoh et al. (2017) failed to observe any change in CBF as measured by Doppler ultrasound. However, they observed an increase in CVR (Ogoh et al., 2017). Compared with the literature, our results showing a decrease in CBF after 5-day DI are consistent with the increase in CVR measured during the first week in simulated microgravity. According to studies performed in hindlimb suspension in rats, the increase in CVR could be a consequence of prolonged vasoconstriction in the first days, in response to the increased blood flow in the brain, due to the HDT position. After several days, the chronic vasoconstriction induced hypertrophy and modifications in the wall of cerebral arteries (Wilkerson et al., 2002). Previous studies have depicted hypertrophy in the media layer, an increase in thickness, an increase in spontaneous tone, and myogenic vasoconstriction of brain arteries mediated by altered secretion of endothelial NO (Geary et al., 1998; Zhang et al., 2001; Wilkerson et al., 2005). According to the authors, the prolonged vasoconstriction and these histological changes could be responsible for an increase in CVR, thus contributing to the decrease in CBF (Wilkerson et al., 2005). During DI5-CUFFS, Robin et al. (2020) observed a decrease in plasma volume by 15–20% throughout DI experiment. Likewise, during 3-day DI, Ogoh et al. (2017) demonstrated a correlation between the decrease in plasma volume and the decrease in blood flow velocity and conductance in the internal carotid artery, suggesting that the loss of plasma volume also contributes to the vasoconstriction of cerebral arteries. In accordance with the literature, we hypothesized that the decrease in rCBF after 5-day DI is the consequence of three mechanisms that all contribute to the increase in CVR: vasoconstriction of cerebral arteries in response to increased CBF induced by the cephalad fluid shift; the decrease in plasma volume; and a moderate increase in ICP, which may contribute to the increase in CVR through compression of cerebral blood vessels.

Cerebral autoregulation is the process of maintaining CBF relatively constant for CPP ranging from 50 to 150mmHg. Above these limits, CBF varies proportionally to CPP (Tameem and Krovvidi, 2013). Cerebral autoregulation is mainly mediated by small arteries that modify their diameter according to the change in CPP, in order to maintain constant CBF (Kontos et al., 1978). Indeed, cerebral autoregulation has been shown to be preserved or even improved in short-term studies (Kermorgant et al., 2020). Nevertheless, according to studies in rats, an increase in ICP may increase CVR through compression of the cerebral blood vessels (Wilkerson et al., 2002).

The basal ganglia interact with the cortex in a system of cortico-subcortical loops, in order to integrate cortical information and relay it to the cortex *via* the thalamus and brainstem (Parent and Hazrati, 1995). As they form the hub of information processing in the brain, these regions may be more intensely affected by change in CBF. An alternative explanation for the greater decrease in rCBF in the basal ganglia concerns the potential modification in neurotransmitter metabolism. Until now, to the best of our knowledge, little is known about neurotransmitter metabolism in humans in microgravity. In a study performed in rats, a change in the binding of neurotransmitters to their receptors was noted after 7days on board Spacelab 3. 5-HT1 receptors were more numerous, and binding of dopamine D-2 in the striatum was decreased (Miller et al., 1989).

### Nonspecific Factors That May Influence rCBF

Many factors could have influence on rCBF. Neurosensory stimulation during injection may influence the HMPAO distribution in the brain (Woods et al., 1991). Thereby, we paid attention to isolate the subjects from neurosensorial stimulation during the injection at BDC-4 and at R0.

Cerebral blood flow changes according to the circadian rhythm. Indeed, it has been showed that CBF velocity is lower in the morning than in the afternoon and in the evening (Conroy et al., 2005). In our study, we performed the HMPAO acquisitions in the morning, roughly at the same hour at BDC-4 and at R0, consequently, the circadian rhythm had little influence on our results.

Hypocapnia is known to reduce CBF by decreasing CPP and decreasing CVR (Grüne et al., 2015). However, breathing function seems to be not altered in DI (Popova et al., 2013).

### No Significant Effect of Thigh Cuffs on rCBF

We did not find any significant change in rCBF after 5-day DI between the cuffs and control groups. We hypothesized that, by limiting the cephalad fluid shift and its consequences, thigh cuffs limited the increase in CVR. During 5-day DI, Arbeille et al. (2020) found a significantly attenuated increase in volume in the right jugular vein (measured with ultrasound) at 2h post-immersion in the cuffs groups (control group: 0.27±0.15cm^3^ to 0.94±0.22cm^3^ *p*<0.05; cuffs group: 0.32±0.13cm^3^ to 0.64±0.32cm^3^; *p*<0.05). However, at DI4, there was no longer any significant difference between the control and cuffs group (control group: 0.47±0.22cm^3^; cuffs group: 0.35±0.14cm^3^, *p*<0.05). Moreover, the right jugular vein was less dilated compared to 2h post-immersion. Therefore, thigh cuffs seemed to be effective in limiting the dilatation of the jugular vein in the first few hours of DI, but their effectiveness seemed to diminish after a few days of DI. Studies suggested that thigh cuffs have an effect against the cephalad fluid shift and its consequences only when they are worn, and that there was no significant memory effect when they were removed at night (Herault et al., 2000). It is worth noting that rCBF was measured in the morning, after a night without thigh cuffs. Therefore, the absence of a significant effect of thigh cuffs on the modification of rCBF in our study has many possible explanations, including a lack of statistical power, the fact that thigh cuffs appear to have little effect on the cephalad fluid shift after 5-day DI, and the absence of a memory effect on rCBF after a night without thigh cuffs.

### Justification for the Choice of ^99m^Tc-HMPAO-SPECT

^99m^Tc-hexamethyl propylene amine oxime SPECT is a tried and tested technique for measuring rCBF (Abdel-Dayem et al., 1988); however, it is not a technique for measuring CBF in large vessels. ^99m^Tc-HMPAO is a SPECT tracer, which has lower spatial resolution than a PET tracer (e.g., ^18^F-FDG, O^15^H_2_). The choice of this tracer was ideal for our study, as ^99m^Tc-HMPAO reaches its binding peak 2–3min after being injected. This allowed us, by injecting participants at the end of DI in the bath, to image rCBF while they were still immersed. After the end of DI, participants underwent a lower-body negative pressure test at the MEDES clinic after the injection, and then went to the nuclear medicine department for the SPECT scans. Because of the study design, the interval between the injection and scans was different at BDC-4 than at R0: scans began 20min after injection at BDC-4, and after 90min at R0. Because of the irreversible brain binding of 99mTc-HMPAO after its injection, we were able to make the acquisitions comparable. We corrected for the difference of acquisition time after injection at BDC-4 and at R0, by calculating a weighting factor for each image, based on the 99mTc decay constant. We then applied this weighting factor, for each image, in the analysis with SPM12 software.

### Study Limitations

Our study had several limitations. Because of the semi-quantitative measurement of rCBF with ^99m^Tc-HMPAO, it is not possible to determine in our study whether the decrease in rCBF was sufficient to induce or be a consequence of brain function impairments. Moreover, it was not planned in the design of the experimentation to collect neurological clinical data, in order to correlate with the rCBF modification.

The small sample size (*N*=18) may have weakened the statistical power of our results. This could explain why we did not find a significant result by correcting the alpha risk for multiple testing by a familywise error rate or false discovery rate. However, we did adjust the alpha risk and applied a good extent threshold that made our results more robust.

For radioprotection reasons, we injected the volunteers, in our initiative, with a less active radiotracer (261±8MBq), compared to the recommended standards (555–1,110MBq). We did not lengthen the time acquisition compared to the recommendations for a reason of comfort for the subjects (30s per projection and 30min for the total time acquisition; Kapucu et al., 2009); that could have weakened the signal-to-noise ratio of our images.

Moreover, DI is a ground-based model of microgravity with a particular environment for the subject, such as physical immobility, that could also influence the decrease in cerebral perfusion. Further studies are needed to explore modifications in CBF in microgravity.

## Conclusion

That is the first study measuring rCBF in DI, we measured a decrease in rCBF in cortical and subcortical regions after a 5-day DI. We hypothesized that prolonged vasoconstriction of cerebral arteries in response to increased CBF resulting from the cephalad fluid shift, the decrease in plasma volume, and a moderate increase in ICP may contribute to the increase in CVR, thus inducing a decrease in rCBF. Although our study has several biases, that could influence the change in rCBF, this study could be considered as an explorative investigation that shows interesting results. Further studies are needed to better understand the effects and consequences of microgravity on rCBF.

## Data Availability Statement

The raw data supporting the conclusions of this article will be made available by the authors, without undue reservation.

## Ethics Statement

The studies involving human participants were reviewed and approved by CPP Est III: 2 October 2018, no. ID RCB 2018-A01470-55. The patients/participants provided their written informed consent to participate in this study. Written informed consent was obtained from the individual(s) for the publication of any potentially identifiable images or data included in this article.

## Author Contributions

M-PB, AB, AP-LT, and PPa conceived and designed the study. LG, M-PB, EC, AB, MB, J-AL, AP-LT, and PPa took part at the experimentation. LG analyzed the data. LG, MK, EC, AP-LT, and PPa drafted the manuscript. All authors contributed to the article and approved the submitted version.

## Funding

This study was supported by CNES (N° 2018 – 4800000970).

## Conflict of Interest

The authors declare that the research was conducted in the absence of any commercial or financial relationships that could be construed as a potential conflict of interest.

## Publisher’s Note

All claims expressed in this article are solely those of the authors and do not necessarily represent those of their affiliated organizations, or those of the publisher, the editors and the reviewers. Any product that may be evaluated in this article, or claim that may be made by its manufacturer, is not guaranteed or endorsed by the publisher.
